# Lactic-fermented egg white improves visceral fat obesity in Japanese subjects—double-blind, placebo-controlled study

**DOI:** 10.1186/s12944-017-0631-2

**Published:** 2017-12-08

**Authors:** Ryosuke Matsuoka, Keiko Kamachi, Mika Usuda, Wei Wang, Yasunobu Masuda, Masaaki Kunou, Akira Tanaka, Kazunori Utsunomiya

**Affiliations:** 1 R&D Division, Kewpie Corporation, 2-5-7 Sengawa Kewport, Sengawa-Cho, Chofu-Shi, Tokyo, 182-0002 Japan; 2 Nutrition Clinic, Kagawa Nutrition University, 3-24-3, Komagome, Toshima-ku, Tokyo, 170-8481 Japan; 3 Department of Internal Medicine, The Jikei University School of Medicine, 3-25-8, Nishi-shinbashi, Minato-ku, Tokyo, 105-8461 Japan

**Keywords:** Egg white, Visceral fat, Obesity, Japanese, Protein

## Abstract

**Background:**

It was reported that egg white protein (EWP) reduced body fat in rats. We developed a lactic-fermented egg white (LE) that facilitates the consumption of egg whites by fermenting them with lactobacillus, and were able to study their intake in humans. In this double-blind, placebo-controlled design, we evaluated the effect of LE on visceral fat area (VFA).

**Methods:**

Participants included 37 adult males and females aged ≥40 years (VFA at navel ≥100 cm^2^). They were divided into two groups: the control group and the LE group. The control and LE groups consumed drinks containing whey and LE, respectively, for 12 weeks (providing 8 g protein/day). VFA was measured at baseline and at week 12 of intake. Abdominal girth was measured at baseline and at weeks 6 and 12.

**Results:**

LE intake decreased VFA significantly compared with baseline (−8.89 cm^2^, *p* < 0.05), and VFA was significantly lower than that in the control group (+1.71 cm^2^, p < 0.05). The LE group showed significant improvement in the ratio of visceral to subcutaneous fat area compared with baseline and the control group (p < 0.05).

**Conclusions:**

The results demonstrated that LE reduces VFA and improves the ratio of visceral to subcutaneous fat area. As other measurement items were not influenced, we concluded that LE improves visceral fat obesity.

**Trial registration:**

This clinical trial was retrospectively registered with the University hospital Medical Information Network (UMIN) Center, (UMIN000026949; registered on 11/04/2017; http://www.umin.ac.jp/).

## Background

Visceral fat obesity causes complications such as dyslipidaemia, abnormal glucose tolerance, and hypertension. Along with serum LDL-cholesterol concentration, it is a risk factor for arteriosclerotic disease [1]. In Japan, approximately 50% men and 20% women ≥40 years have metabolic syndrome or pre-metabolic syndrome, which is becoming more serious [2].

Hen eggs contain almost all essential nutrients (except vitamin C and dietary fiber), and are known to be of high nutritive value [3]. Attention has been drawn to the cholesterol contained in eggs, and nutritional education for dyslipidaemia recommends avoiding egg consumption. Although the cholesterol in eggs is found in the yolk, some reports have indicated that consumption of egg yolk does not increase blood cholesterol levels [4–6] or mortality due to coronary heart disease [7]. Therefore, we considered whether the constituent that improves lipid metabolism is contained in eggs, specifically egg white protein (EWP). EWP is high in protein and low in fat and has been reported to reduce serum total and LDL cholesterol levels [8–10].

EWP is said to lower blood cholesterol by inhibiting cholesterol absorption in the small intestines. However, in doing so, triglyceride absorption is also inhibited [11]. Therefore, EWP not only reduces serum LDL cholesterol level but is also believed to prevent arteriosclerotic disease by reducing visceral fat.

In an experiment on rats, Matsuoka et al. reported that egg white proteins could reduce body and abdominal fat [12]. However, it is unclear whether the reduction was effective in human.

The consumption of egg white itself poses a problem in terms of taste and physical properties. We therefore developed a lactic-fermented egg white (LE) that facilitates the consumption of egg whites by fermenting them with lactobacillus, and were able to study their intake in humans [13, 14]. In a previous study, it was reported that the EWPs ovalbumin and ovotransferrin could inhibit lipid absorption [11]. It has been reported that beta-conglycinin, a soy protein, reduced visceral fat at the speed of 5 g/day [15]. Ovalbumin and ovotransferrin are reported to contain 54% and 11% EWP, respectively [16]. If we suppose that ovalbumin and ovotransferrin have the same visceral fat-reducing effect as beta-conglycinin at a daily intake of 5 g, a daily intake of 8 g of EWP would be required. We therefore conducted a double-blind, placebo-controlled study to evaluate the visceral fat-reducing effect of protein in Japanese adults with visceral fat obesity, via daily intake of LE for 12 weeks.

## Methods

### Test foods

The control drink and the drink containing LE were prepared by Co-op Foods Co., Ltd. (Tokyo, Japan). Flavors, sweeteners and water were added to the whey (Nippon-Shinyaku Co., Ltd., Kyoto, Japan) and LE [13] (Kewpie Egg Corporation, Tokyo, Japan) of each drink. Once homogeneously dispersed, the preparation was heat-sterilized. Measurement using the Kjeldahl method [17] indicated that both drinks contained 8 g of protein per 180 g serving. Ovalbumin content in LE was assessed by the sandwich ELISA procedure using anti-chicken ovalbumin polyclonal antibody, and horseradish peroxidase-labeled anti-chicken ovalbumin polyclonal antibody, respectively. A commercial kit, Egg (Ovalbumin) ELISA kit II (Morinaga Institute of Biological Science Inc., Yokohama, Japan) was used with absorbance detected at 450 nm by a multi-detection microplate reader (Powerscan® HT, DS Pharma Biomedical Co. Ltd., Osaka, Japan). Ovalbumin content of the LE drink was 4.17 g/180 g, as measured via sandwich ELISA. Based on a previous report that ovotransferrin accounts for 11% of total EWP [17], the content of ovotransferrin was calculated to be 0.88 g/180 g LE (Calculated value).

### Participants and test methods

Recruitment was done per the following process: Japanese subjects with body mass index (BMI) (≥25), aged more than 40 years (male) or postmenopausal female, who were not undergoing treatment for hyperlipidemia or diabetes, who had no subjective symptoms of gout and who were capable of filling out the required forms, such as self-diagnosis forms, and of visiting a designated institution as scheduled. The exclusion criteria were as follows: taking drugs that could potentially affect the test results (e.g., anti-hyperlipidemic agents, anti-diabetic agents, oral corticosteroid formulations, and antihypertensive agents); regular consumption of foods for specified health uses that could potentially affect test results; excessive alcohol consumption; suspected allergic reactions (particularly to egg and milk); participation in other clinical studies; a history of serious liver damage, kidney damage, or myocardial infarction; a history of, or current, hepatitis; and serious anemia. In this study, 80 adults were screened and 48 individuals with visceral fat area (VFA) (≥100 cm^2^) were included as test subjects.

The study followed a double-blind, parallel-arm design. Participants were divided into two groups: a control group and LE group. The control group was given a drink containing whey (8 g of protein), and the LE group was given a drink containing LE (8 g of protein) every day at breakfast for 12 continuous weeks.

At 0 and 12 weeks of intake, computed tomographic (CT) scanning of the abdomen (Aquilion™, Toshiba Medical Systems Corporation, Tochigi, Japan) was performed, and VFA was measured at the navel (Fat Scan, East Japan Institute of Technology Co., Ltd., Ibaraki, Japan). CT scans were conducted at the Nakajima Clinic (Tokyo, Japan). At weeks 0, 6, and 12, bodyweight, blood pressure, and abdominal girth, respectively, were measured after fasting overnight. Blood was sampled from a forearm vein, and blood tests were performed at baseline, and weeks 6 and 12 of intake.

### Blood tests

Peripheral blood test was performed using flow cytometry. Serum analysis included the following items: total cholesterol (enzyme method); HDL cholesterol (direct method); triglyceride (enzyme method); free fatty acid (FFA; enzyme method); phospholipid (enzyme method); glucose (hexokinase UV test); HbA1c (latex coagulating method); RLP cholesterol (immunoadsorption); insulin (CLEIA method); AST (JSCC transferable method); ALT (JSCC transferable method); gamma-GTP (JSCC transferable method); blood urea nitrogen (BUN; urease LED UV method); creatinine (enzyme method); and uric acid (enzyme method). LDL cholesterol concentration was calculated using the Friedewald formula [18]. Blood tests were performed by SRL Corporation (Tokyo, Japan).

### Dietary intake

The nutritional value of the content of meals recorded by participants over a three-day period was calculated using Excel Eiyoukun Food Frequency Questionnaire version 5 (Kenpakusha, Tokyo, Japan). The 2010 Standard Table of Food Composition in Japan database was used in this nutrition-calculation software.

### Statistical analysis

Test results are shown as mean ± standard error. Comparisons with baseline values were performed using paired *t*-tests for VFA and Dunnett’s test for all other items. Comparisons with the control group were performed using the student’s *t*-test. A significance level of less than 5% was considered substantial. Statistical analyses were performed using SPSS version 20 (SPSS Inc., Tokyo, Japan). Change in VFA is indicated by ∆ cm^2^.

## Results

### Adherence and participant attributes

**Fig. 1:**
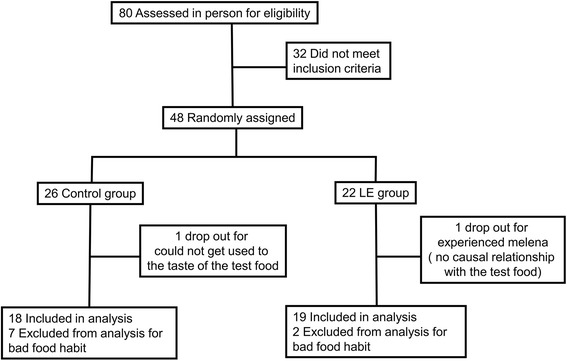
Participant flow through a randomized trial

During the study period, two participants withdrew from the study; one individual in the control group could not get accustomed to the taste of the test food and one individual in the LE group experienced melena. The latter’s condition was assumed to be due to the participant’s tendency toward constipation and the melena was thus determined to not be causally related to the test food (Fig. 1).

From the control group, seven participants were excluded: two of whom did not fast prior to blood sampling; four who did not complete the food diary sufficiently to track actual food intake; and one who was inconsistent in the timing of test food consumption. From the LE group, two participants were excluded: one who did not fast prior to blood sampling and one who completed the food diary insufficiently (Fig.1). From the above, we analyzed 18 individuals in the control group and 19 in the LE group. There were no significant differences observed at baseline between the two groups in terms of age, height, or BMI (*p* > 0.05, Table 1).

**Table 1:** Background of subjects

	Control			LE		
Age (y)	55.2	±	1.5	56.6	±	1.2
Height (cm)	166	±	3	167	±	2
Body weight (kg)	76.1	±	2.5	78.4	±	2.2
BMI	27.5	±	0.7	28.0	±	0.7
Systolic blood presure (mmHg)	131	±	5	135	±	4
Diastolic blood pressure (mmHg)	80.1	±	3.1	87.1	±	2.5

Mean ± SE of 18 (control) and 19 (LE)

*Control* Control group, *LE* Lactic fermented egg white group

### Dietary intake

There was no significant change in the amount of food consumed during the study period in either group (Table 2).

**Table 2:** Dietary intake of subjects

		Intake Period (Weeks)								
		0			6			12		
Energy (kcal)	Control	1956	±	109	2073	±	163	1976	±	112
	LE	2163	±	175	1978	±	93	2012	±	77
Protein (g)	Control	71.9	±	5.3	75.1	±	5.2	73.4	±	5.0
	LE	80.1	±	6.9	73.0	±	3.3	74.2	±	3.3
Lipids (g)	Control	64.9	±	4.9	68.6	±	7.2	68.0	±	4.6
	LE	68.7	±	8.5	61.0	±	4.2	66.8	±	5.2
Carbohydrate (g)	Control	251	±	15	266	±	21	250	±	15
	LE	276	±	20	244	±	13	251	±	11
Cholesterol (mg)	Control	388	±	47	396	±	42	391	±	37
	LE	330	±	33	335	±	27	351	±	36
Dietary fiber (g)	Control	11.5	±	0.9	12.4	±	1.1	11.8	±	0.9
	LE	13.6	±	1.3	12.1	±	1.3	12.7	±	0.8

Mean ± SE of 18 (control) and 19 (LE)

*Control* Control group, *LE* Lactic fermented egg white group

### Physical condition

The abdominal girth of the control group was significantly lower at 12 weeks than at baseline. No significant difference was observed for any other items between the control and LE groups, nor compared to baseline (Table 3).

**Table 3:** Results of body composition at the navel level

		Intake Periods (Weeks)					
		0			12		
Body weight (kg)	Control	76.1	±	2.5	76.0	±	2.5
	LE	78.4	±	2.2	78.5	±	2.2
BMI	Control	27.5	±	0.7	27.4	±	0.7
	LE	28.0	±	0.7	28.1	±	0.7
Waist (cm)	Control	100	±	2	99.8	±	2.0^a^
	LE	99.3	±	1.9	98.4	±	2.2
Total fat area (cm^2^)	Control	391	±	22	391	±	27
	LE	386	±	27	390	±	28
Subcutaneous fat area (cm^2^)	Control	249	±	20	246	±	24
	LE	240	±	24	254	±	23^a^
Visceral fat area (cm^2^)	Control	143	±	7	144	±	8
	LE	145	±	9	136	±	9^a^
Visceral/Subcutaneous	Control	0.766	±	0.078	0.597	±	0.052
	LE	0.656	±	0.048	0.546	±	0.041*

Mean ± SE of 18 (control) and 19 (LE)

^a^p < 0.05 vs. 0 weeks by paired t-test

*Control* Control group, *LE* Lactic fermented egg white group

### Fat area at the navel

There was no significant difference in the total fat area in the LE group compared to baseline or the control group (Table 3). Although no significant difference was observed between the two groups in terms of VFA and subcutaneous fat area, the LE group showed significantly less VFA, and a significant increase in subcutaneous fat area compared to baseline (*p* < 0.05). The control group showed no significant difference compared to baseline (Table 3).

Calculation of the visceral to subcutaneous fat ratio indicated a significantly lower ratio in the LE group compared to baseline. There was no significant difference in the control group compared to baseline or between the two groups (Table 3).

**Fig. 2:**
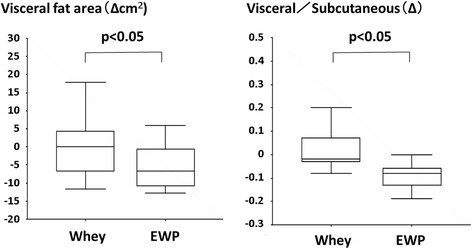
Change in visceral fat area and ratio of visceral to subcutaneous fat area of the subjects fed LE or whey for 12 weeks. Δ: 12 weeks–0 weeks. Mean ± SE of 18 (control) and 19 (LE), *: *p* < 0.05 vs. 0 weeks by paired *t*-test

**Fig. 3:**
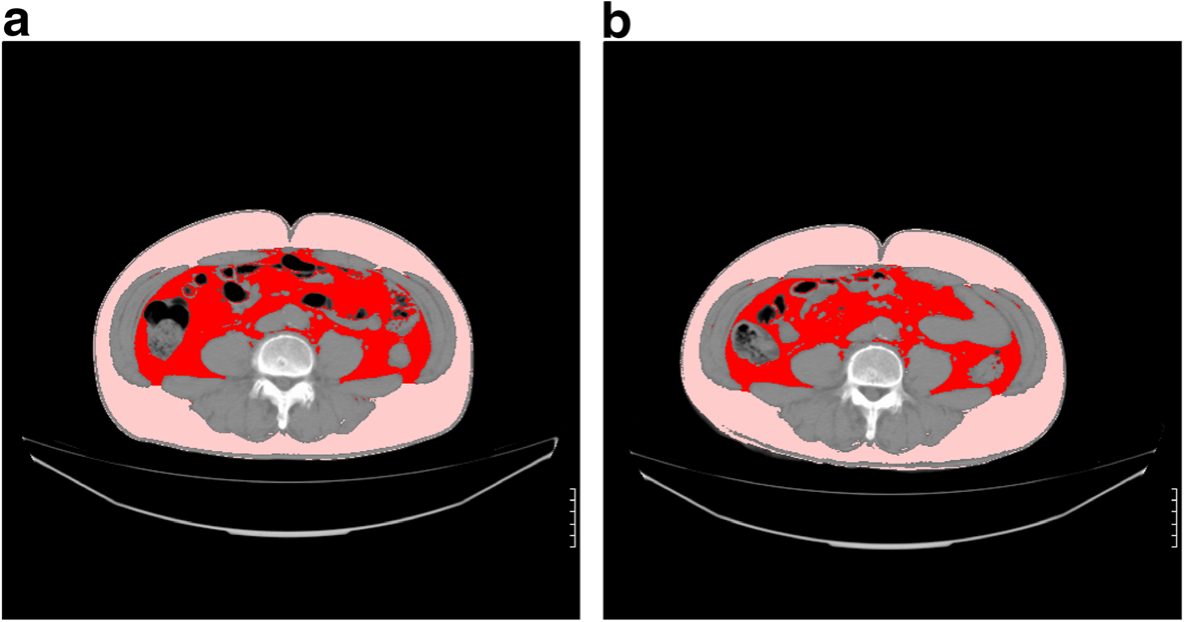
A photographic example of the change in visceral fat area (VFA) of the subjects fed LE for 12 weeks. Red: Visceral fat, Pink: Subcutaneous fat. **a**: Pre-intake (VFA = 119 cm^2^), **b**: Post-intake (VFA = 102 cm^2^)

Calculation of the change in VFA revealed a significant reduction in the LE group compared to both baseline and the control group. With regards to the change in visceral to subcutaneous fat ratio at the navel (∆), the value was significantly reduced by the intake of LE compared to the control group and baseline ratio (Fig. 2 and Fig. 3).

### Serum biochemical analysis

There was no significant variation observed in serum total cholesterol, LDL cholesterol, RLP cholesterol, triglycerides, phospholipids, insulin, or blood glucose levels. While the LE group exhibited significantly lower FFA concentrations compared to the control group in week 6 (*p* < 0.05), there was no significant difference observed in week 12. Moreover, while the LE group showed significantly lower HDL-cholesterol levels in week 6 compared to baseline (*p* < 0.05), there was no significant difference observed in week 12 (Table 4).

**Table 4:** Serum biochemical analysis

		Intake Period (Weeks)									
		0			6			12			
Total Cholesterol	Control	209	±	6	210	±	7	209	±	8	
(mg/dL)	LE	215	±	6	210	±	6	217	±	6	
HDL-Cholesterol	Control	55.8	±	2.26	55.1	±	2.60	54.8	±	2.26	
(mg/dL)	LE	58.1	±	2.85	52.4	±	2.02^a^	55.8	±	2.51	
LDL-Cholesterol	Control	133	±	4	131	±	5	131	±	6	
(mg/dL)	LE	132	±	5	130	±	6	135	±	5	
Triglyceride	Control	127	±	16	150	±	25	123	±	15	
(mg/dL)	LE	132	±	18	150	±	18	134	±	12	
NEFA	Control	496	±	31	553	±	36	515	±	34	
(mEq/L)	LE	481	±	42	443	±	37#	543	±	49	
Phospholipid	Control	218	±	7	221	±	8	215	±	7	
(mg/dL)	LE	224	±	6	221	±	5	222	±	5	
Glucose	Control	92.8	±	2.0	91.7	±	2.0	96.0	±	3.0	
(mg/dL)	LE	92.7	±	1.8	94.4	±	2.1	93.6	±	2.6	
Insulin	Control	6.65	±	0.98	7.32	±	0.95	6.93	±	0.55	
(mg/mL)	LE	6.26	±	0.74	8.35	±	1.33	7.50	±	0.82	
HbA1c (NGSP)	Control	5.53	±	0.09	5.50	±	0.08	5.51	±	0.09	
(%)	LE	5.56	±	0.08	5.54	±	0.10	5.56	±	0.10	
RLP-Cholsterol	Control	4.81	±	0.66	6.25	±	1.29	4.73	±	0.49	
(mg/dL)	LE	5.58	±	0.93	6.73	±	1.21	5.34	±	0.54	

Mean ± SE of 18 (control) and 19 (LE)

^a^p < 0.05 vs. 0 weeks by Dunnett test、#p < 0.05 vs. Control group by Student’s *t*-test

*Control* Control group, *LE* Lactic fermented egg white group

### Hematological tests

The control group exhibited significantly high mean corpuscular volume (MCV) in week 6 compared to baseline, whereas mean corpuscular hemoglobin level (MCHC) in weeks 6 and 12 were significantly lower than baseline (*p* < 0.05). The LE group showed significantly higher MCV in week 6 compared to baseline (p < 0.05; Table 5). Throughout the entire study period, MCV and MCHC were significantly lower in the LE group compared to the control group (p < 0.05). All items’ values stayed within the normal range and no particular problems were observed (Table 5).

**Table 5:** Results of hematological tests and hepatic and kidney function

		Intake Period (Weeks)								
		0			6			12		
WBC	Control	5683	±	334	6072	±	382	5972	±	327
(/μL)	LE	5779	±	310	5758	±	301	5579	±	290
RBC	Control	473	±	10	470	±	9	476	±	10
(/μL)	LE	488	±	11	480	±	10	491	±	9
Hb	Control	14.7	±	0.3	14.7	±	0.3	14.6	±	0.3
(g/dL)	LE	14.8	±	0.3	14.7	±	0.3	14.7	±	0.3
Ht	Control	44.4	±	0.9	44.9	±	0.8	44.6	±	0.8
(%)	LE	45.0	±	0.9	44.8	±	0.8	45.0	±	0.7
MCV	Control	93.9	±	0.6	95.7	±	0.8^a^	93.9	±	0.7
(fl)	LE	92.3	±	0.7	93.5	±	0.8^a^	91.8	±	0.7#
MCH	Control	31.1	±	0.3	31.2	±	0.3	30.8	±	0.3
(pg)	LE	30.3	±	0.3	30.6	±	0.3^a^	30.1	±	0.3*
MCHC	Control	33.0	±	0.2	32.6	±	0.2^a^	32.8	±	0.2
(%)	LE	32.8	±	0.2	32.7	±	0.2	32.7	±	0.1
Platelet	Control	23.8	±	1.4	23.0	±	0.9	22.7	±	0.9
(/μL)	LE	23.7	±	1.3	22.5	±	1.2	22.7	±	1.2
AST	Control	21.9	±	1.8	22.1	±	2.0	24.4	±	3.8
(IU/L)	LE	22.1	±	1.3	24.7	±	1.9	24.9	±	2.3
ALT	Control	27.9	±	4.2	27.2	±	4.2	30.8	±	7.1
(IU/L)	LE	25.0	±	2.1	39.8	±	3.2	28.0	±	2.5
γ-GTP	Control	49.2	±	12.2	48.0	±	11.1	42.4	±	9.1
(IU/L)	LE	42.7	±	9.2	44.3	±	8.9	40.6	±	7.3
BUN	Control	14.6	±	0.9	15.6	±	0.7	14.5	±	0.7
(mg/dL)	LE	13.8	±	0.9	15.9	±	0.8^a^	15.2	±	0.7
Cr	Control	0.737	±	0.024	0.758	±	0.025	0.736	±	0.027
(mg/dL)	LE	0.839	±	0.032#	0.849	±	0.030#	0.853	±	0.035#
UA	Control	6.23	±	0.28	6.56	±	0.28	6.17	±	0.30
(mg/dL)	LE	6.32	±	0.24	6.49	±	0.23	6.18	±	0.24

Mean ± SE of 18 (control) and 19 (LE)

^a^p < 0.05 vs. 0 weeks by Dunnett test、#p < 0.05 vs. Control by Student’s *t*-test

*Control* Control group, *LE* Lactic fermented egg white group

### Liver and kidney functions indices

While the LE group exhibited significantly higher BUN levels in week 6 compared to baseline (p < 0.05), BUN was within the normal range, with no significant difference observed in week 12. The LE group showed significantly lower creatinine levels than the control group at baseline and in week 12 (p < 0.05). However, there was no significant variation observed throughout the intake period, and because changes were within the normal range, this was not considered to pose any problem. For all other items, no influence of the test foods was observed (Table 5).

## Discussion

Results of this study indicated that when 8 g of LE (i.e., EWP) was consumed daily for 12 weeks, VFA was significantly reduced compared to baseline and the control group. Therefore, we concluded that LE has a visceral fat-reduction effect. It has been previously reported that the visceral fat-reduction food constituents include catechine [19] and polyphenol [20]. The degree by which these constituents reduced visceral fat of the food consumed for 12 weeks was −10.3 cm^2^ for catechine and −7.9 cm^2^ for polyphenol, compared to a reduction of 8.89 cm^2^ by LE in this study. This finding suggests that LE has the same effect as food constituents that are generally considered to reduce visceral fat. It has been reported that protein-derived constituents also reduce visceral fat, and therefore, in this study we evaluated the effect of a daily intake of 8 g of lactic acid-fermented albumin as protein, based on a report on beta-conglycinin in soybeans [13, 14]. However, this study did not examine whether less than 8 g of EWP would exhibit a visceral fat-reduction effect and this requires further study. Furthermore, it has been reported that lactoferrin (LF), found in milk protein, has a visceral fat-reduction effect [21]. It has been found that a daily intake of 300 mg of LF significantly reduced VFA compared to baseline and the control group. In this study, the control group was given whey, which contains LF. It was expected that a daily intake of 8 g of whey would correspond to a daily intake of 450 mg of LF and was therefore expected to have a visceral fat-reduction effect. However, whey did not reduce visceral fat in the control group. A possible explanation is that whey contains many nutrients other than LF, which may have competed. It has previously been reported that a daily intake of 28 g of whey had no effect on visceral fat [22]. It was reported that it needs to be subjected to enteric coating to exert its visceral fat–reducing effects because LF itself is vulnerable to heating and is easily degraded by gastric acid [21]. This should explain why the whey sample used in the present study failed to reduce visceral fat.

Furthermore, in this study the ratio of visceral to subcutaneous fat was significantly reduced after consumption of LE compared to baseline ratio and the control group. The visceral to subcutaneous fat ratio is an indicator of visceral fat obesity, with a ratio > 0.4 indicating visceral fat obesity [23]. In our study, LE not only reduced VFA but also considerably reduced the visceral to subcutaneous fat ratio; therefore, we concluded that the symptoms of visceral fat obesity had reduced.

Although it is unclear why LE intake reduces visceral fat, it has been reported that EWP, the main constituent of LE, has an inhibitory effect on the absorption of triglycerides [11]. EWP inhibited the absorption of fat, thereby functioning to reduce lipid content within bile acid micelles. The components involved in this process are ovalbumin and ovotransferrin, both of which reduce lipid content within bile acid micelles to an equivalent extent. No other components have been shown to affect this process [11]. As such, we inferred that these two components might also have come into play in this experiment. Especially, the underlying cause of this has been reported to involve lipase inhibitory activity in ovalbumin [24], the main constituent of EWP, as well as the inhibitory effect of lipid micelles [11]. Furthermore, ovalbumin has been found to form FFAs and gels [25, 26]. Therefore, it is believed that EWP can inhibit the absorption of lipids by way of its physicochemical properties.

EWP has an amino acid score of 100, with a higher rate of net protein utilization than soy and milk proteins [27]. In a previous examination of the effect of EWP on carcass protein contents in rat compared to casein, we reported that EWP showed significantly higher levels than casein [12]. As the main constituent of muscle is protein, it is believed that the increase in carcass protein contents in rats were due to increased muscle mass. In general, however, a muscle does not increase without exercise; thus, further studies should be conducted to determine whether a muscle increases because of the high net protein utilization and increased body protein. The fact that muscle burns fat in the event of increased muscle may be one explanation for the observed reduction in visceral fat.

It is generally believed that active food constituents that reduce visceral fat are often those whose structures do not change through absorption or metabolic process (e.g., polyphenol), the effects of which are easily evaluated in vivo. In contrast, proteins become effective after being broken down into peptides and amino acids in the digestive tract. With regards to the details of the active constituents contained in EWP, we believe that the visceral fat-reduction mechanism should first be clarified and then confirmed via in vitro evaluation.

In this study, participants consumed either LE or whey drink at breakfast. Suzuki et al. reported that pre-exercise intake of EWP increases muscle and muscular strength [28]. It has been reported that consuming hen’s eggs at breakfast induced satiety and reduced participants’ body fat [29]. However, in this study, while participants’ energy intake hardly changed and the lactic acid drink did not affect their satiety, we confirmed that visceral fat was reduced. On the contrary, protein intake following muscle strength training is recommended to increase muscles [30]. Therefore, we believe that consuming LE after dinner or before sleeping might reduce visceral fat more effectively. Furthermore, if the visceral fat-reduction mechanism of EWP lies in the inhibition of lipid absorption [11], it may be preferable to consume LE at dinnertime, the meal with the highest energy intake. Moreover, previous research has reported that the circadian rhythm gene BMAL1, which is involved in lipid accumulation [31], is abundantly secreted at night. Therefore, we believe that the consumption of LE at dinnertime could more effectively inhibit the accumulation of lipids. Per these factors, while the present study indicated that LE intake at breakfast reduces VFA, the timing at which it effectively reduces visceral fat was not clarified and should be examined in future studies.

In the present study, the blood biomarkers exhibited no significant differences between week 0, week 6, and week 12. Although the present study involved subjects with slightly high levels of visceral fat weight, the other parameters such as blood lipids were within the normal range. This might have resulted in no significant changes being observed in the blood biomarkers. In previous studies, subjects with slightly high levels of serum cholesterol showed significant decreases in serum total cholesterol and LDL-cholesterol concentrations at week 4 and week 8 during 8 weeks of LE consumption as compared with prior to consumption [8]. This suggests the possibility that marked improvements may be achieved if subjects with borderline or abnormal blood biomarker values are involved.

Concern has been raised that EWP intake could cause allergies and biotin deficiency. In this study, individuals with food allergies were excluded during recruitment. It has been demonstrated that avidin found in raw egg whites binds to biotin, thereby reducing its absorption [32]. While the participants of this study were asked to consume 8 g of EWP per day for 12 consecutive weeks, there was no sign of clinical symptoms. A study in which participants consumed 200 g of dried egg white (approximately 160 g of EWP) daily reported signs of biotin deficiency after week 7 [33, 34]. The consumption period was 12 weeks; however, the amount of EWP intake was 8 g, which was 1/20th of the amount of EWP that caused biotin deficiency in the previous study, and may explain the observed lack of biotin deficiency. Furthermore, when egg white is in its raw state or heated, it has been reported that electrophoretic avidine bands disappear upon activation of pepsin [34]. During the digestive process, as pepsin is usually activated, we believe that a daily intake of 8 g of LE for 12 weeks should not cause biotin deficiency. Finally, we measured markers of liver and kidney function. No abnormal increases or decreases in numeric values were observed, suggesting that EWP can be consumed safely.

Metabolic syndrome, triggered by obesity, leads to hypertension, abnormal glucose tolerance, and is ultimately considered a cause of arteriosclerotic diseases. If we pursue this study further and clarify the visceral fat-reduction effect of EWP, we believe that it could improve the quality of life of patients with obesity. Furthermore, if the practical application of LE, which facilitates the egg white intake, becomes available, we believe that it would provide a good source of protein while reducing visceral fat and might be utilized to promote the health among the Japanese.

## Conclusions

The results demonstrated that LE reduces VFA and improves the ratio of visceral to subcutaneous fat area. As other measurement items were not influenced, we concluded that LE improves visceral fat obesity.

## Abbreviations

BMI: Body Mass Index
CT: Computed tomographic
EWP: Egg white protein
LE: Lactic-fermented egg white
VFA: Visceral fat area
